# Supplementary material for: Estimated incidence and number of new cases with prenatal alcohol exposure and fetal alcohol spectrum disorders in Finland 1990–2025
Source: PLoS One. 2026 Oct 21;21(10):e0349964. doi: 10.1371/journal.pone.0349964 (PMC13686515; doi:10.1371/journal.pone.0349964)
Supplement: Peer review history

# Peer review: Decision Letter 0

**DOI:** 10.1371/journal.pone.0349964.r001

**Authors:** Ricardo Cobucci

18 Jun 2026

Dear Dr. Jolma,

Please submit your revised manuscript by Aug 17 2026 11:59PM. If you will need more time than this to complete your revisions, please reply to this message or contact the journal office at plosone@plos.org. When you're ready to submit your revision, log on to https://www.editorialmanager.com/pone/ and select the 'Submissions Needing Revision' folder to locate your manuscript file.

- A letter that responds to each point raised by the academic editor and reviewer(s). You should upload this letter as a separate file labeled 'Response to Reviewers'.
- A marked-up copy of your manuscript that highlights changes made to the original version. You should upload this as a separate file labeled 'Revised Manuscript with Track Changes'.
- An unmarked version of your revised paper without tracked changes. You should upload this as a separate file labeled 'Manuscript'.

If you would like to make changes to your financial disclosure, please include your updated statement in your cover letter. Guidelines for resubmitting your figure files are available below the reviewer comments at the end of this letter.

If applicable, we recommend that you deposit your laboratory protocols in protocols.io to enhance the reproducibility of your results. Protocols.io assigns your protocol its own identifier (DOI) so that it can be cited independently in the future. For instructions see: https://journals.plos.org/plosone/s/submission-guidelines#loc-laboratory-protocols. Additionally, PLOS ONE offers an option for publishing peer-reviewed Lab Protocol articles, which describe protocols hosted on protocols.io. Read more information on sharing protocols at https://plos.org/protocols?utm_medium=editorial-email&utm_source=authorletters&utm_campaign=protocols.

As the corresponding author, your ORCID iD is verified in the submission system and will appear in the published article. PLOS supports the use of ORCID, and we encourage all coauthors to register for an ORCID iD and use it as well. Please encourage your coauthors to verify their ORCID iD within the submission system before final acceptance, as unverified ORCID iDs will not appear in the published article. *Only*  the individual author can complete the verification step; PLOS staff *cannot*  verify ORCID iDs on behalf of authors.

We look forward to receiving your revised manuscript.

Kind regards,

Ricardo Ney Oliveira Cobucci, Ph.D

Academic Editor

PLOS One

Journal Requirements:

When submitting your revision, we need you to address these additional requirements.

1. Please ensure that your manuscript meets PLOS ONE's style requirements, including those for file naming. The PLOS ONE style templates can be found at

https://journals.plos.org/plosone/s/file?id=wjVg/PLOSOne_formatting_sample_main_body.pdf and

https://journals.plos.org/plosone/s/file?id=ba62/PLOSOne_formatting_sample_title_authors_affiliations.pdf

2. We note that the grant information you provided in the ‘Funding Information’ and ‘Financial Disclosure’ sections do not match.

When you resubmit, please ensure that you provide the correct grant numbers for the awards you received for your study in the ‘Funding Information’ section.

3. If the reviewer comments include a recommendation to cite specific previously published works, please review and evaluate these publications to determine whether they are relevant and should be cited. There is no requirement to cite these works unless the editor has indicated otherwise.

[Note: HTML markup is below. Please do not edit.]

Reviewers' comments:

Reviewer's Responses to Questions

**Comments to the Author**

1. Is the manuscript technically sound, and do the data support the conclusions?

Reviewer #1: Yes

Reviewer #2: Partly

**********

2. Has the statistical analysis been performed appropriately and rigorously?

Reviewer #1: Yes

Reviewer #2: No

**********

3. Have the authors made all data underlying the findings in their manuscript fully available?

The PLOS Data policy

Reviewer #1: Yes

Reviewer #2: Yes

**********

4. Is the manuscript presented in an intelligible fashion and written in standard English?

Reviewer #1: Yes

Reviewer #2: Yes

**********

Reviewer #1: It is a well written manuscript, and I appreciate the overall goal of the manuscript. The authors are attempting to estimate births with PAE/FASD across time and conclude PAE/FASD in Finland is concerning and more objective studies are needed. I agree this is important.

Introduction:

• Please include the definition of binge (what quantity) in the past 30 days.

• Please include the definition of ‘heavy PAE’ as reported by WHO GHO portal.

Methods:

• The strongest finding reported is Pa(t). This is likely the absolute lower bound for what the true estimate of pregnancies with PAE can be. I suggest the authors consider highlighting this finding more.

• It is not entirely justified why biomarker based-results were assigned a double weight in the estimates of Ph(t). In the estimation of �, the authors state “(�) was formed by combining self-reported heavy use data before year 2004 with biomarker data after year 2004.” Therefore, ��is not solely based on biomarkers.

• It would be interesting to see how the estimates changes if Ph(t) was = (N(t)-R(t))x alpha. By simplifying the estimates, the reader is better able to gauge how much all of the various estimations (and assumption) that were made as part of the analytic process may be inflating or deflating the results. For example, defining Aa as the mean prevalence of the two age groups. It is likely that either group is over/under- represented in terms of which group accounted for more live births across time. Alcohol consumption, at least heavy alcohol consumption, tends to decrease with age. What effect does using the average have? Or another assumption of setting the missing 1990 Ah to the average of Ah values, is it justifiable?

• Did the authors consider not using a weighting system for diagnostic system in the country multiplier? Could the authors conduct separate analyses for each diagnostic system?

• I appreciate the authors’ desires to attempt to capture cases within international adaptions and immigration. However, I feel the assumptions made regarding FASD prevalence is not well justified, with the exception of Eastern Europe which has been determined in the literature. One might suggest that Ethiopia is more similar to South Africa, than South Africa is to Eastern Europe, so why are different assumptions being made? Likewise, why the assumption that 20% of adoptees from Colombia, Philippines, and Thailand but assuming other countries have negligible prevalence among adoptees? I would suggest the authors consider either providing estimates both with and without the inclusion of Ia(t) and Ii(t), or remove it altogether. If both estimates are kept in, please highlight the speculative nature of these estimates.

Results:

• Please ensure that all figures can stand alone. Please define models & equations within the figures as well as in the text.

• Figure 2A is difficult to read with the overlapping symbols and linear fit/CI. Consider having the linear fit and 95CI on a separate part of the Figure 2B and rename current 2B to 2C.

• Figure 3B & 4B – consider adding ‘%’ sign to the y axis label.

• Figure 5B reports new FASD cases yet the written text in the manuscript describes percentages. Please make consistent with % or counts.

• Line 387, it is unclear how the “approximately 1500-2500 new children with FASD are estimated annually” were derived. It does not appear to match the counts reported in Figure 5B or any other results provided.

• If keeping the international estimates, consider adding a total column to that table.

Discussion:

• The authors state that “the results of the present study also depend on several assumptions”. This is true. Please expand to highlight other major assumptions made.

• Consider commenting on the diagnostic capabilities, or the lack of, and the need for clinical providers to make such a diagnosis. Why are so few children being identified in the national register of congenital malformations. Not diagnosed because did not want to label for stigma or another reason? Not diagnosed because clinical staff uncomfortable with making the diagnosis? No clinicians at all? Families not seeking diagnosis?

• Consider commenting on the public health implication. Even without ‘true’ prevalence numbers from active case ascertainment studies, what is the actionable prevention/intervention take home for Finland now.

Reviewer #2: Overall comments:

This study uses a mathematical modelling approach to estimate PAE and FASD incidence in Finland from 1990-2025. While any PAE levels were found to substantially decrease over this time period, likely due to changes to national recommendations supporting abstinence during pregnancy in 2004, there was only a modest decline in heavy PAE. Modelled FASD incidence showed a similar, modest decrease, irrespective of the model used.

This study addresses an important gap (i.e. prevalence estimates for PAE and FASD in Finland) and is methodologically transparent. While there was the need for a heavy reliance on assumptions and data extrapolation, due to limited data specifically from Finland, this was acknowledged and discussed by the authors. Also, the heterogenous nature of the data (i.e. predominantly non-anonymous self-report, biomarkers, requirement for data sources from international studies) was duly recognised.

The PAE levels and FASD incidence, while higher than previous estimates, are plausible and comparable to other countries with similar lifestyles to Finland.

Specific comments:

• Some of the figures are a little unclear. In particular, Figure 2A could benefit from colouration of the 4 variables and trend lines or graphing separately and using the same axes scales.

• Given the large number of assumptions that needed to be made, perhaps the authors could consider sensitivity analyses in their modelling. This could investigate how changes to the estimated variables and constants using a range (as well as using the mean) could impact the final prevalence estimates.

• The abstract refers to a ‘multiplier model’ and a ‘PAE-based model’ but then these terms were not used elsewhere in the manuscript. Instead, the authors described these as ‘Model M’ and ‘Model L’ in the methods and results. Please change how these are referenced for consistency.

• Please explain further why the non-significant (at the typical p<0.05 level) correlation reported at line 364 was used as the basis for the multiplier model. What implications are there on the model’s robustness/accuracy to estimate FASD prevalence?

• Can the authors please comment on their use of linear extrapolation of the data? In some cases, the data appears to be non-linear (e.g. Figure 2B, as noticed by the authors).

**********

what does this mean?). If published, this will include your full peer review and any attached files.

If you choose “no”, your identity will remain anonymous but your review may still be made public.

**Do you want your identity to be public for this peer review?**  For information about this choice, including consent withdrawal, please see our Privacy Policy

Reviewer #1: No

Reviewer #2: No

**********

[NOTE: If reviewer comments were submitted as an attachment file, they will be attached to this email and accessible via the submission site. Please log into your account, locate the manuscript record, and check for the action link "View Attachments". If this link does not appear, there are no attachment files.]

To ensure your figures meet our technical requirements, please review our figure guidelines: https://journals.plos.org/plosone/s/figures

You may also use PLOS’s free figure tool, NAAS, to help you prepare publication quality figures: https://journals.plos.org/plosone/s/figures#loc-tools-for-figure-preparation.

NAAS will assess whether your figures meet our technical requirements by comparing each figure against our figure specifications.

# Author comment: Author response to Decision Letter 1

**DOI:** 10.1371/journal.pone.0349964.r002

10 Aug 2026

Dear Dr. Oliveira Cobucci,

Thank you for the opportunity to improve our work. To this end, we found the reviewers’ comments both helpful and encouraging. We have edited the text accordingly and we are pleased to present our revised manuscript. Our responses to the specific comments are presented below.

Mirjami Jolma and coauthors

Editor’s notes

1. Please ensure that your manuscript meets PLOS ONE's style requirements, including those for file naming. The PLOS ONE style templates can be found at

https://journals.plos.org/plosone/s/file?id=wjVg/PLOSOne_formatting_sample_main_body.pdf and

https://journals.plos.org/plosone/s/file?id=ba62/PLOSOne_formatting_sample_title_authors_affiliations.pdf

--Our response: We have corrected the references to figures and renamed the figure files as described in the formatting guidelines.

2. We note that the grant information you provided in the ‘Funding Information’ and ‘Financial Disclosure’ sections do not match.

When you resubmit, please ensure that you provide the correct grant numbers for the awards you received for your study in the ‘Funding Information’ section.

--Our response: The funding information includes now grant number and URL of the funder (Emil Aaltonen foundation).

Reviewer #1

It is a well written manuscript, and I appreciate the overall goal of the manuscript. The authors are attempting to estimate births with PAE/FASD across time and conclude PAE/FASD in Finland is concerning and more objective studies are needed. I agree this is important.

--Our response: We thank the reviewer for the positive view towards our work.

Introduction:

• Please include the definition of binge (what quantity) in the past 30 days.

--Our response: We thank the reviewer for this helpful suggestion. We have revised the introduction to define heavy episodic (binge) drinking as the consumption of at least 60 grams of pure alcohol on a single occasion, consistent with the WHO definition (lines 63 – 65). We have also added the IOM diagnostic threshold for PAE sufficient to support an FASD diagnosis (lines 59 – 61).

• Please include the definition of ‘heavy PAE’ as reported by WHO GHO portal.

--Our response: We would like to clarify that the WHO GHO portal does not report “heavy PAE”. Instead, it reports the prevalence of heavy episodic (binge) drinking among women aged ≥15 years, defined as the proportion who consumed ≥60 grams of pure alcohol on at least one occasion during the past 30 days. This is the WHO indicator (variant Aₕ) used in our study, and we have revised the description in the Methods (Table 1. Parameters) to make this definition explicit.

Methods:

• The strongest finding reported is Pa(t). This is likely the absolute lower bound for what the true estimate of pregnancies with PAE can be. I suggest the authors consider highlighting this finding more.

--Our response: We thank the reviewer for this insightful suggestion. We agree that P_a (t) represents a key finding of our study and is likely to reflect the absolute lower bound of the true incidence of PAE. We have revised the Methods to clarify this interpretation (lines 177 – 179) and have emphasized its importance in the Discussion (lines 387 – 392).

• It is not entirely justified why biomarker based-results were assigned a double weight in the estimates of Ph(t). In the estimation of α, the authors state “(α) was formed by combining self-reported heavy use data before year 2004 with biomarker data after year 2004.” Therefore, α is not solely based on biomarkers.

--Our response: We thank the reviewer for highlighting this point. We agree that the original wording was unclear and could have led to the misunderstanding that α was solely based on biomarker data. This was not our intention. The estimate of α was derived by combining biomarker-based data with anonymous self-reported data. The double weighting of α reflects our assumption that these data sources provide a more reliable estimate of heavy alcohol use during pregnancy than non-anonymous self-reported data, which are prone to underreporting. We have revised the text to clarify the derivation of α and the rationale for the applied weighting (lines 145 –147 and 181 – 189).

• It would be interesting to see how the estimates changes if Ph(t) was = (N(t)-R(t))x alpha. By simplifying the estimates, the reader is better able to gauge how much all of the various estimations (and assumption) that were made as part of the analytic process may be inflating or deflating the results. For example, defining Aa as the mean prevalence of the two age groups. It is likely that either group is over/under- represented in terms of which group accounted for more live births across time. Alcohol consumption, at least heavy alcohol consumption, tends to decrease with age. What effect does using the average have? Or another assumption of setting the missing 1990 Ah to the average of Ah values, is it justifiable?

--Our response: We thank the reviewer for noting this omission. We have now included sensitivity analyses to the manuscript (lines 294 – 301). Besides testing the effects of various variables, we also compare the suggested simplified model to the “full model”. The results are shown as part of the Results section (lines 381 – 386), and the analyses are included in the R source file.

• Did the authors consider not using a weighting system for diagnostic system in the country multiplier? Could the authors conduct separate analyses for each diagnostic system?

--Our response: We thank the reviewer for this relevant suggestion. We evaluated the impact of diagnostic system weighting in the country multiplier by performing a sensitivity analysis without these weights. This resulted in a 6% change in the estimated FASD incidence, indicating that this assumption had a limited influence on the overall estimates (lines 381 – 386). Separate analyses by diagnostic system were considered, but the number of available studies using individual diagnostic criteria was limited, resulting in too few data points for reliable separate modeling.

• I appreciate the authors’ desires to attempt to capture cases within international adaptions and immigration. However, I feel the assumptions made regarding FASD prevalence is not well justified, with the exception of Eastern Europe which has been determined in the literature. One might suggest that Ethiopia is more similar to South Africa, than South Africa is to Eastern Europe, so why are different assumptions being made? Likewise, why the assumption that 20% of adoptees from Colombia, Philippines, and Thailand but assuming other countries have negligible prevalence among adoptees? I would suggest the authors consider either providing estimates both with and without the inclusion of Ia(t) and Ii(t), or remove it altogether. If both estimates are kept in, please highlight the speculative nature of these estimates.

--Our response: We thank the reviewer for this important comment. We agree that the assumptions used to estimate FASD among internationally adopted and immigrant children were highly uncertain due to very limited research on the subject, and that these estimates require a more comprehensive analysis. Although these groups appear to be overrepresented among clinically diagnosed FASD cases in Finland and are therefore of clinical relevance, the available data are insufficient for reliable prevalence estimation by country of origin. Because contribution of these groups to the overall population estimates was relatively small and their inclusion introduced additional uncertainty, we have removed the estimates of FASD related to immigration and international adoption from the current manuscript. We have retained a brief discussion of this issue as an area requiring further research (lines 484 – 488).

Results:

• Please ensure that all figures can stand alone. Please define models & equations within the figures as well as in the text.

--Our response: We thank the reviewer for this suggestion. Model definitions have now been added to the legends of Figures 3, 4 and 5 to allow the figures to be interpreted independently.

• Figure 2A is difficult to read with the overlapping symbols and linear fit/CI. Consider having the linear fit and 95CI on a separate part of the Figure 2B and rename current 2B to 2C.

--Our response: We agree. Figure has now been reformatted according to the reviewer’s suggestion to improve readability.

• Figure 3B & 4B – consider adding ‘%’ sign to the y axis label.

--Our response: Percentage signs have been added to the y-axis labels of Figures 3B and 4B.

• Figure 5B reports new FASD cases yet the written text in the manuscript describes percentages. Please make consistent with % or counts.

--Our response: We have revised to report counts consistent with Figure 5B to the text (lines 354 – 363) and also include a Figure 5C to show percentages.

• Line 387, it is unclear how the “approximately 1500-2500 new children with FASD are estimated annually” were derived. It does not appear to match the counts reported in Figure 5B or any other results provided.

--Our response: We have replaced the visually approximated numbers with exact estimates derived from the models (lines 378 – 379).

• If keeping the international estimates, consider adding a total column to that table.

--Our response: We have removed the immigration-related estimates from the models due to the uncertainty associated with very limited data (see response above).

Discussion:

• The authors state that “the results of the present study also depend on several assumptions”. This is true. Please expand to highlight other major assumptions made.

--Our response: We thank the Reviewer for this important suggestion. We agree that several assumptions underlying the modeling approach should be highlighted more explicitly. We have now expanded the Limitations section to describe the major assumptions (467 – 478).

• Consider commenting on the diagnostic capabilities, or the lack of, and the need for clinical providers to make such a diagnosis. Why are so few children being identified in the national register of congenital malformations. Not diagnosed because did not want to label for stigma or another reason? Not diagnosed because clinical staff uncomfortable with making the diagnosis? No clinicians at all? Families not seeking diagnosis?

--Our response: We thank the reviewer for this important comment. We agree that the current discrepancy between the estimated and diagnosed cases requires discussion. Underdiagnosis of FASD is a well-recognized global problem. The current data do not allow us to determine specific reasons for underdiagnosis. However, international research has identified several contributing factors. We have expanded the Discussion to highlight these factors (lines 439 – 445).

• Consider commenting on the public health implication. Even without ‘true’ prevalence numbers from active case ascertainment studies, what is the actionable prevention/intervention take home for Finland now.

--Our response: We thank the reviewer for this suggestion. We agree that the public health implications should be emphasized. We have expanded the Discussion and Conclusion to highlight this (lines 446 – 453, 491 – 501).

Reviewer #2

Overall comments:

This study uses a mathematical modelling approach to estimate PAE and FASD incidence in Finland from 1990-2025. While any PAE levels were found to substantially decrease over this time period, likely due to changes to national recommendations supporting abstinence during pregnancy in 2004, there was only a modest decline in heavy PAE. Modelled FASD incidence showed a similar, modest decrease, irrespective of the model used.

This study addresses an important gap (i.e. prevalence estimates for PAE and FASD in Finland) and is methodologically transparent. While there was the need for a heavy reliance on assumptions and data extrapolation, due to limited data specifically from Finland, this was acknowledged and discussed by the authors. Also, the heterogenous nature of the data (i.e. predominantly non-anonymous self-report, biomarkers, requirement for data sources from international studies) was duly recognised.

The PAE levels and FASD incidence, while higher than previous estimates, are plausible and comparable to other countries with similar lifestyles to Finland.

--Our response: We thank the reviewer for the positive evaluation of our work and for recognizing both the importance of the research question and the transparency of our modeling approach. We appreciate the reviewer’s thoughtful assessment and constructive comments, which have helped us to improve the manuscript.

Specific comments:

• Some of the figures are a little unclear. In particular, Figure 2A could benefit from colouration of the 4 variables and trend lines or graphing separately and using the same axes scales.

--Our response: Figure 2 has now been reformatted, as suggested.

• Given the large number of assumptions that needed to be made, perhaps the authors could consider sensitivity analyses in their modelling. This could investigate how changes to the estimated variables and constants using a range (as well as using the mean) could impact the final prevalence estimates.

--Our response: We thank the reviewer for this suggestion. We have now included sensitivity analyses to evaluate the impact of the main model assumption on the estimated prevalence. The methods and principal results have been added to the main text (lines 294 – 301 and 381 – 386, respectively) and the full set of analyses is provided in the supplementary R file.

• The abstract refers to a ‘multiplier model’ and a ‘PAE-based model’ but then these terms were not used elsewhere in the manuscript. Instead, the authors described these as ‘Model M’ and ‘Model L’ in the methods and results. Please change how these are referenced for consistency.

--Our response: We have revised the terminology throughout the manuscript so that the abstract and the main text use consistent model names.

• Please explain further why the non-significant (at the typical p<0.05 level) correlation reported at line 364 was used as the basis for the multiplier model. What implications are there on the model’s robustness/accuracy to estimate FASD prevalence?

--Our response: We thank the reviewer for this important comment. The linear correlation was used to derive the country multiplier rather than to test a statistical hypothesis. Because only nine eligible prevalence studies were available, the analysis had limited statistical power and thus the correlation did not reach conventional statistical significance. We have clarified this point in the manuscript and emphasized that the limited number of studies increase the uncertainty of the multiplier model (lines 348 – 351). This uncertainty is also reflected in the results of sensitivity analyses (lines 381 – 386) and is discussed as a limitation (lines 475 – 478).

• Can the authors please comment on their use of linear extrapolation of the data? In some cases, the data appears to be non-linear (e.g. Figure 2B, as noticed by the authors).

--Our response: We thank the reviewer for this comment. We agree that some variables may have non-linear temporal trends. However, due to the limited temporal data nonlinear models resulted in implausible fluctuations and were considered more prone to overfitting. We therefore used linear modeling as a conservative approach to estimate temporal trends. We have clarified this rationale on the Methods (lines 144 – 146) and need for additional biomarker-based studies in the Figure 2B legend.

Attachment Submitted filename: Response to reviewers_20260808.docx

# Peer review: Decision Letter 1

**DOI:** 10.1371/journal.pone.0349964.r003

**Authors:** Ricardo Cobucci

14 Sep 2026

Estimated incidence and number of new cases with prenatal alcohol exposure and fetal alcohol spectrum disorders in Finland 1990-2025

PONE-D-26-22232R1

Dear Dr. Jolma,

We’re pleased to inform you that your manuscript has been judged scientifically suitable for publication and will be formally accepted for publication once it meets all outstanding technical requirements.

Within one week, you’ll receive an e-mail detailing the required amendments. When these have been addressed, you’ll receive a formal acceptance letter and your manuscript will be scheduled for publication.

An invoice will be generated when your article is formally accepted. Please note, if your institution has a publishing partnership with PLOS and your article meets the relevant criteria, all or part of your publication costs will be covered. Please make sure your user information is up-to-date by logging into Editorial Manager at Editorial Manager® and clicking the ‘Update My Information' link at the top of the page. For questions related to billing, please contact billing support.

If your institution or institutions have a press office, please notify them about your upcoming paper to help maximize its impact. If they’ll be preparing press materials, please inform our press team as soon as possible -- no later than 48 hours after receiving the formal acceptance. Your manuscript will remain under strict press embargo until 2 pm Eastern Time on the date of publication. For more information, please contact onepress@plos.org.

Kind regards,

Ricardo Ney Oliveira Cobucci, Ph.D

Academic Editor

PLOS One

Additional Editor Comments (optional):

Reviewers' comments:

Reviewer's Responses to Questions

**Comments to the Author**

Reviewer #1: (No Response)

Reviewer #2: All comments have been addressed

**********

2. Is the manuscript technically sound, and do the data support the conclusions?

Reviewer #1: Yes

Reviewer #2: Yes

**********

3. Has the statistical analysis been performed appropriately and rigorously?

Reviewer #1: Yes

Reviewer #2: Yes

**********

4. Have the authors made all data underlying the findings in their manuscript fully available?

The PLOS Data policy

Reviewer #1: Yes

Reviewer #2: Yes

**********

5. Is the manuscript presented in an intelligible fashion and written in standard English?

Reviewer #1: Yes

Reviewer #2: Yes

**********

Reviewer #1: The authors addressed all of my previous comments well. Two very minor comments remain.

1. Please remove the sentence "With decreasing birth rates and increasing immigration, migration was incorporated to account for children with FASD born abroad who later immigrated, either through international adoptions or with their birth parents."

2. Please define the eight-pointed star in the Figure legend of Figure 3.

Reviewer #2: (No Response)

**********

what does this mean?). If published, this will include your full peer review and any attached files.

If you choose “no”, your identity will remain anonymous but your review may still be made public.

**Do you want your identity to be public for this peer review?**  For information about this choice, including consent withdrawal, please see our Privacy Policy

Reviewer #1: No

Reviewer #2: No

**********

# Editor report: Acceptance letter

**DOI:** 10.1371/journal.pone.0349964.r004

**Authors:** Ricardo Cobucci

PONE-D-26-22232R1

PLOS One

Dear Dr. Jolma,

I'm pleased to inform you that your manuscript has been deemed suitable for publication in PLOS One. Congratulations! Your manuscript is now being handed over to our production team.

At this stage, our production department will prepare your paper for publication. This includes ensuring the following:

* All references, tables, and figures are properly cited

* All relevant supporting information is included in the manuscript submission,

* There are no issues that prevent the paper from being properly typeset

You will receive further instructions from the production team, including instructions on how to review your proof when it is ready. Please keep in mind that we are working through a large volume of accepted articles, so please give us a few days to review your paper and let you know the next and final steps.

Lastly, if your institution or institutions have a press office, please let them know about your upcoming paper now to help maximize its impact. If they'll be preparing press materials, please inform our press team within the next 48 hours. Your manuscript will remain under strict press embargo until 2 pm Eastern Time on the date of publication. For more information, please contact onepress@plos.org.

You will receive an invoice from PLOS for your publication fee after your manuscript has reached the completed accept phase. If you receive an email requesting payment before acceptance or for any other service, this may be a phishing scheme. Learn how to identify phishing emails and protect your accounts at https://explore.plos.org/phishing.

If we can help with anything else, please email us at customercare@plos.org.

Thank you for submitting your work to PLOS One and supporting open access.

Kind regards,

PLOS One Editorial Office Staff

on behalf of

PROFESSOR Ricardo Ney Oliveira Cobucci

Academic Editor

PLOS One
